# The Role of CARD9 Deficiency in Neutrophils

**DOI:** 10.1155/2021/6643603

**Published:** 2021-01-04

**Authors:** Ruanmei Sheng, Xiaoming Zhong, Zhiwen Yang, Xuemin Wang

**Affiliations:** ^1^Songjiang Hospital Affiliated to Shanghai Jiao Tong University School of Medicine (Preparatory Stage), Shanghai, China; ^2^Shanghai Songjiang Clinical Medical College of Nanjing Medical University, Shanghai, China; ^3^Jiangxi Province Tumor Hospital, Nanchang, China

## Abstract

Neutrophils play a critical role in innate immune defense and directly contribute to infectious and autoimmune ailments. Great efforts are underway to better understand the nature of neutrophilic inflammation. Of note, CARD9, a myeloid cell-specific signaling protein that mainly expresses in macrophages and dendritic cells, is also present in neutrophils, emerging as a critical mediator for intercellular communication. CARD9–deficiency neutrophils display an increased susceptibility to fungal infection that primarily localize to the central nervous system, subcutaneous, and skin tissue. Additionally, CARD9–deficiency neutrophils are associated with some autoimmune diseases and even provide protection against a few bacteria. Here, the review summarizes recent preclinical and clinical advances that have provided a novel insight into the pathogenesis of CARD9 deficiency in neutrophils.

## 1. Introduction

CARD9 is a central integrator of innate and adaptive immunity that is mainly expressed in myeloid cells, especially in macrophages and dendritic cells. CARD9 as an intracellular adaptor molecule could activate NF–*κ*B and/or MAPK signal pathways in macrophages and dendritic cells, leading to an inflammatory cascade against the invasive bacteria, fungi, and virus [1, 2].

Neutrophils are known as the most abundant circulating leukocytes, participating in the organization of the antimicrobial host defense, autoimmune, and aseptic inflammatory processes. Emerging evidence demonstrates that CARD9 is not only present in dendritic cells and macrophages but also in neutrophils, exhibiting a similar role in fungal infection, bacterial infection, and autoimmune diseases [3–5]. Different from the previously published paper on CARD9 deficiency in macrophages, myeloid, and dendritic cells, CARD9–deficiency neutrophils may greatly impair neutrophil functions, leading to fungal brain infection, arthritis, and dermatitis [3–5]. What is more, a novel mechanism that negative feedback regulation of Dok3-PP1 complex was found in neutrophils [6]. Thus, this review is to discuss the most recent findings on the pathogenesis of CARD9 in neutrophils, while the function of CARD9 in macrophages and dendritic cells is omitted here.

## 2. CARD9-Deficient Neutrophils and Fungal Infection

### 2.1. Fungal Brain Infection

Some findings have demonstrated that CARD9 deficiency gives rise to susceptibility to invasive fungal infection in the brain. In the central nervous system (CNS) of CARD9-deficient patients, significant eosinophil infiltration and defective neutrophil accumulation were observed [3, 4, 7–9]. Neutrophils act as critical mediators of host defenses against systemic *C. albicans* infection, whereas eosinophils do not play this role [10]. The phenomenon of CNS-specific neutropenia may be attributed to the deficiency of neutrophil-targeted chemoattractants. In the infected cerebrospinal fluid (CSF) of CARD9-deficient patients, a striking lack of neutrophil-targeted chemokines, namely, CXCL1, CXCL2, CXCL5, and IL-8, was detected [4, 11]. Conversely, the CSF of CARD9^+/+^ patients with *C. albicans* meningitis exhibited significant induction of CXC neutrophil-recruiting chemokines [4, 11]. Furthermore, microglial deletion of CARD9 in the brain greatly reduced the neutrophil-recruitment of IL-1*β* and CXCL1 [11]. These data suggested that CARD9 plays an important role in the production of neutrophil-specific chemoattractant factors, promoting the accumulation of neutrophils at the infection site. Therefore, CARD9 deficiency might predispose patients to CNS infection as a result of CNS-specific neutropenia.

A total of three patients with CARD9 deficiency who suffer from *Candida albicans* meningitis have been reported, showing an intrinsic functional impairment in neutrophils [3–5]. CARD9-deficient neutrophils harvested from the three patients displayed a selective defect in fungal clearance.

A 13-year-old Asian girl was diagnosed with *Candida dubliniensis* meningoencephalitis [3]. As a result of c.214G>A and c.1118G>C mutations in the CARD9 gene, the patient showed a complete absence of CARD9 protein in neutrophils. CARD9-deficient neutrophils isolated from the patient's peripheral blood displayed a specific killing defect for unopsonized *C. albicans* yeasts, but not opsonized *C. albicans* yeasts. Surprisingly, the *ex vivo* impaired killing capacity of CARD9-deficient neutrophils against unopsonized *Candida* yeasts could be effectively restored upon opsonization with human serum, potentially explaining the susceptibility of patients to *Candida* infection in the CNS [3]. Because of the blood-brain barrier, CNS parenchyma has limited access to plasma opsonization [12].

An 11-year-old girl, from North Carolina, USA, was reported with *C. albicans* infection of the CNS [4]. In this case, a c.170G>A CARD9 missense mutation induced the generation of full-length CARD9 protein, with similar CARD9 protein expression levels observed between the patient and healthy donor neutrophils; however, this mutation resulted in the impaired production of proinflammatory factors. Next, the study investigated the antifungal ability of CARD9-deficient neutrophils, indicating a selective killing defect against *C. albicans* yeasts rather than hyphae. In addition, CARD9-deficient neutrophils exhibited normal intrinsic cellular functions, including their transmigration from the blood into the infected brain and cellular survival *in vivo*.

A 4-year-old Turkish girl was hospitalized for chronic *C. albicans* meningitis [5]. The patient was reported to possess a homozygous point mutation in exon 6 (c.883C>T) of CARD9, which led to a lack of CARD9 protein in the patient's neutrophils. The killing capacity of CARD9-deficient neutrophils was obviously impaired against nonopsonized *C albicans* yeasts, but functioned normally against serum-opsonized yeasts. The defective neutrophil function observed in the absence of serum opsonin might predispose patients to chronic *C. albicans* infection of the CNS where opsonization is naturally low.

### 2.2. Fungal Subcutaneous Infection

CARD9 deficiency in patients has been linked to subcutaneous phaeohyphomycosis caused by *Phialophora verrucosa* [13, 14]. The collected four patients presented with persistent red plaques and nodules on their cheeks and faces, showing a slight improvement and/or limited effect upon receiving antifungal drug therapy. In these patients, CARD9 deficiency exhibited a striking decrease of IL-17 and IL-22 in serum, impaired the differentiation of TH17 innate immune cells, and subsequently led to the patients' suppressed antifungal immunity. Neutrophils isolated from CARD9-deficient patients showed marked downregulation of IL-8, TNF-*α*, and IL-6 inflammatory factors upon *P. verrucosa* stimulation. In addition, CARD9^˗/˗^ neutrophils exhibited a selective defect in the killing of unopsonized *P. verrucosa* conidia, but their ability to kill *P. verrucosa* hyphae remained intact. The impaired fungal killing capacity in CARD9^−/−^ neutrophils could be completely restored in the presence of human serum. In view of the low level of serum opsonization in the subcutaneous tissue, CARD9 in human neutrophils was indispensable for the immune response to subcutaneous *P. verrucosa* infection.

### 2.3. Fungal Extrapulmonary and Pulmonary Infections

CARD9 deficiency in patients constituted an increasing susceptibility to extrapulmonary and pulmonary fungal infections [15–17]. For these patients, CARD9^−/−^ neutrophils exhibited normal intrinsic chemotaxis from the blood into the *Aspergillus*-infected tissue, and intact killing capacity, phagocytosis, and the induction of an oxidative burst upon fungus-specific stimulation [16, 17]. Interestingly, CARD9-deficient neutrophils showed an impaired killing capacity against *Aspergillus fumigatus*, including opsonized or unopsonized conidia and hyphae [17]. This differed from the previously reported selective defect in eliminating unopsonized *C. albicans* yeasts. Therefore, CARD9 in human neutrophils is indispensable for intraabdominal and pulmonary *Aspergillus* infections.

### 2.4. Molecular Mechanisms of CARD9-Deficient Neutrophils in Fungal Infections

**Figure 1:**
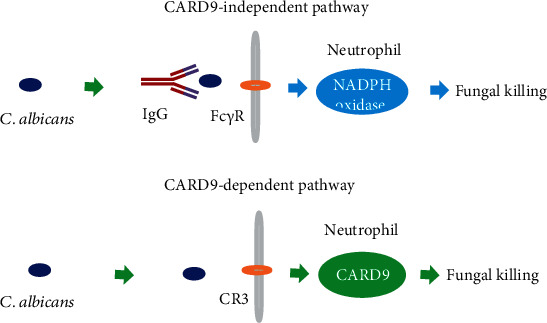
Neutrophils kill fungus via CARD9-dependent or independent mechanisms. Neutrophil-mediated killing mechanisms, either via CR3 or the Fc*γ* receptors, depended on fungal recognition. The killing capacity of CARD9-deficient neutrophils could be restored by opsonization of *C. albicans* with human IgG antibody.

In general, CARD9-deficiency patients greatly impaired the neutrophil functions, showed an inhibited secretion of inflammatory factor, and further resulted in the patients' suppressed antifungal immunity, and eventually induced fungal infection in the CNS. In this study, Drewniak et al. uncovered the underlying mechanisms for eliminating nonopsonized *C. albicans* from patient's neutrophils [3]. First, CARD9-deficient neutrophils displayed appropriate intrinsic functions, with a completely normal capacity to generate reactive oxygen species, NADPH oxidase activity, adhesion, chemotaxis, degranulation of azurophilic and specific granules, and only a slight decrease in IL-8 release in response to various pathogens. Second, the phagolysosomes of CARD9-deficient neutrophils were found to contain more *Candida* yeasts than the control neutrophils, showing a significantly enlarged appearance. Neutrophil production in the phagolysosomes was induced by the increased osmotic pressure resulting from the breakdown of microbial proteins after intracellular killing [18]. Recently, Gazendam et al. discovered that human neutrophils kill invading *C. albicans* through two independent and distinct mechanisms [19]. The first mechanism for unopsonized *C. albicans* clearance was dependent on complement receptor 3 (CR3) recognitions and then triggering the downstream signaling including spleen-tyrosine kinase (Syk), phosphatidylinositol-3-kinase (PI3K), and CARD9, but independent of NADPH oxidase activation. The second mechanism for serum-opsonized *C. albicans* clearance depended on Fc*γ* receptors, protein kinase C, and reactive oxygen species production by the NADPH oxidase system, but was independent of CARD9 signaling. Briefly, the presence of two independent pathways for fungal killing depended on the recognition of either unopsonized or opsonized *C. albicans* by human serum or IgG (Figure 1). Due to the low level of IgG opsonization in the fungal-infected CSF [20], the CARD9-dependent CR3-Syk-PI3K pathway played a critical role in eliminating nonopsonized C. albicans, leading to susceptibility to CNS fungal disease in the absence of CARD9 in neutrophils. These findings provided further insight into the intrinsic functional defects in neutrophils in CNS-specific fungal infections.

**Figure 2:**
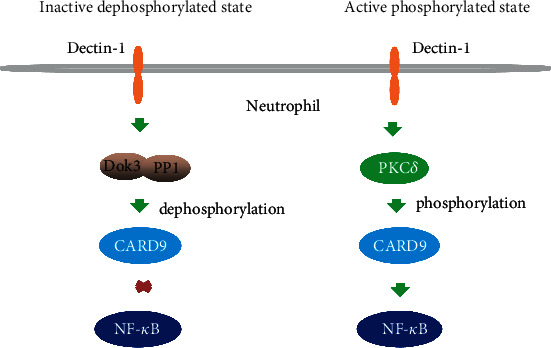
Phosphorylated and dephosphorylated states of CARD9. In a resting state, Dok3 and PP1 combined with CARD9 in neutrophils, maintaining the inactive dephosphorylated state of CARD9. Upon *Candida* infection, the Dok3-PP1 complex dissociated from CARD9, and CARD9 was subsequently phosphorylated by PKC*δ*.

CARD9-mediated signaling drives an inflammatory circuit that triggers an excessive inflammatory response [21]. After CARD9 phosphorylation at Thr231 by PKC*δ*, CARD9 protein interacts directly with Bcl-10 to form the signaling complex. The CARD9/Bcl-10 complex induces the activation of NF-*κ*B and MAPKs and the subsequent release of inflammatory cytokines (Figure 2).

This study provided novel mechanistic insight into CARD9-induced inflammatory signaling [6]. In neutrophils, the downstream of kinase 3 (Dok3) was found to interact with protein phosphatase 1 (PP1), bringing PP1 into the vicinity of CARD9, which maintained CARD9 protein dephosphorylation in an inactive state. Upon dissociation of the Dok3-PP1 complex from CARD9, CARD9 was phosphorylated by PKC*δ*, and NF-*κ*B was subsequently activated [6]. Thereby, Dok3 and PP1 were regarded as critical regulators of CARD9 dephosphorylation, negatively regulating CARD9 activity in neutrophils (Figure 2).

## 3. CARD9-Deficient Neutrophils and Bacterial Infection

*Moraxella catarrhalis*, a Gram-negative bacterium, is a common cause of chronic obstructive pulmonary disease (COPD). Neutrophil numbers in the patient's respiratory tract are found to increase significantly, triggering antimicrobial activity.

The membrane surface of human neutrophils displays a distinct carcinoembryonic antigen-related cell adhesion molecule 3 (CEACAM3) that can specifically bind to ubiquitous surface protein A1 (UspA1) of *M. catarrhalis.* After UspA1-CEACAM3 interactions, the CARD9 adaptor molecule was activated, leading to a significant increase in the production of reactive oxygen species, granulocyte degranulation, NF-*κ*B, and IL-8 in neutrophils. Conversely, CARD9 deficiency in neutrophils resulted in significant inhibition of the NF-*κ*B inflammatory signaling pathway upon stimulation with *M. catarrhalis* [22].

Because of their short cellular lifespan, it is not currently possible to investigate the molecular mechanisms of action of human neutrophils using *in vitro* RNA interference. Instead, an acute promyelocytic leukemia-derived cell line NB4 was used as an *in vitro* cellular model in this study. To provide evidence for the CARD9-mediated CEACAM3-NF-*κ*B pathway, CARD9^−/−^ neutrophils from patients infected with *M. catarrhalis* need to be further investigated in the future [22].

## 4. CARD9-Deficient Neutrophils and Autoimmune Diseases

In addition to its antimicrobial function, CARD9 has also been associated with human aseptic diseases including inflammatory bowel disease [23], rheumatoid arthritis [24], IgA nephropathy [25], and ankylosing spondylitis [26]. As reported previously [3, 4], CARD9 is not only present in dendritic cells and macrophages but also in neutrophils. Although CARD9 in dendritic cells and macrophages is confirmed to be involved in aseptic inflammation [27], its functions in neutrophils remain unclear.

CARD9 knockdown effectively alleviated autoantibody-induced arthritis and dermatitis in a mouse model [28]. CARD9-deficient neutrophils isolated from mice showed a normal respiratory burst and degranulation, but an obvious impairment in NF-*κ*B activation, proinflammatory gene expression, and the release of various chemokines and cytokines. Furthermore, the CARD9-mediated signaling pathway in neutrophils was initiated via C-type lectin receptors in a receptor-proximal Src-family/Syk/PLCg2 module, leading to NF-*κ*B activation and cytokine release through the CARD9/Bcl10/Maltl complex.

Because CARD9 is expressed in dendritic cells, macrophages, and neutrophils, it is difficult to dissect the inflammatory activity of CARD9-deficient neutrophils in patients and mice. To resolve this issue, mice specifically lacking CARD9 in the neutrophil compartment (referred to as CARD9^*Δ*PMN^ mice) were used to study autoantibody-induced arthritis and dermatitis, along with complete CARD9 knockout mice (referred to as CARD9^−/−^ mice). Compared with CARD9^−/−^ mice, CARD9^*Δ*PMN^ mice showed the same protection from *in vivo* inflammatory reactions, indicating that CARD9 in neutrophils, instead of macrophages and dendritic cells, was required for the autoantibody-induced inflammatory response. This was the first direct evidence that CARD9 deficiency in neutrophils plays an important role in suppressing *in vivo* inflammation [28].

CARD9 knockdown was also shown to ameliorate neutrophilic dermatosis in mice [29]. After *in vitro* incubation with recombinant murine IL-1*α*, CARD9 deficiency in neutrophils significantly dampened MAPK and NF-*κ*B inflammatory signaling. Consequently, CARD9 in neutrophils may trigger an excessive inflammatory response in the mouse model of neutrophilic dermatosis.

## 5. Conclusions

CARD9 plays a critical role in innate immune defense. This review focuses on the effects of CARD9 deficiency on the host immune response in neutrophils. Of note, CARD9^*Δ*PMN^ mutant mice, with a lineage-specific deletion of CARD9 in their neutrophils, provide compelling evidence that CARD9 in neutrophils acts as a critical contributor to *in vivo* inflammation. However, the biological functions of CARD9 in neutrophils remain elusive. Further work will be required to determine whether the impaired killing capacity of CARD9-deficient neutrophils is a major contributor to fungal infection in these patients, how CARD9-deficient neutrophils operate their organ-specific functions against fungal infection in the CNS and skin, and why CARD9-deficient neutrophils exhibit a selective defect in fungal killing predisposing the host to *C. albicans* and *P. verrucosa* infection but not *Aspergillus* infection.
